# AttSCNs: A Bayesian-Optimized Hybrid Model with Attention-Guided Stochastic Configuration Networks for Robust GPS Trajectory Prediction

**DOI:** 10.3390/e27111094

**Published:** 2025-10-23

**Authors:** Xue-Bo Jin, Ye-Qing Wang, Jian-Lei Kong, Yu-Ting Bai, Ting-Li Su

**Affiliations:** School of Computer and Artificial Intelligence, Beijing Technology and Business University, Beijing 100048, China; jinxuebo@btbu.edu.cn (X.-B.J.); 2330601022@st.btbu.edu.cn (Y.-Q.W.);

**Keywords:** trajectory prediction, stochastic configuration networks, attention mechanism, GPS tracking, Bayesian hyperparameter optimization, Internet of Vehicles

## Abstract

Trajectory prediction in the Internet of Vehicles (IoV) is crucial for enhancing road safety and traffic efficiency; however, existing methods often fail to address the challenges of colored noise in GPS data and long-term dependency modeling. To overcome these limitations, this paper proposes AttSCNs, a probabilistic hybrid framework integrating stochastic configuration networks (SCNs) with an attention-based encoder to model trajectories while quantifying prediction uncertainty. The model leverages SCNs’ stochastic neurons for adaptive noise filtering, attention mechanisms for dependency learning, and Bayesian hyperparameter optimization to infer robust configurations as a posterior distribution. Experimental results on real-world GPS datasets (10,000+ urban/highway trajectories) demonstrate that AttSCNs significantly outperform conventional approaches, reducing RMSE by 36.51% compared to traditional SCNs and lowering MAE by 97.8% compared to Kalman filter baselines. Moreover, compared to the LSTM model, AttSCNs achieve a 52.5% reduction in RMSE and a 68.5% reduction in MAE, with real-time inference speed. These advancements position AttSCNs as a robust, noise-resistant solution for IoV applications, offering superior performance in autonomous driving and smart city systems.

## 1. Introduction

Accurate vehicle trajectory prediction is a cornerstone of the Internet of Vehicles (IoV), enabling key applications such as intelligent route planning, traffic flow optimization, and autonomous driving [1]. By leveraging GPS data, trajectory prediction aims to forecast future vehicle movements in highly complex and dynamic traffic conditions. Nevertheless, the effectiveness of conventional models is often constrained by inherent challenges in real-world GPS data, including temporally correlated (colored) noise, long-term dependencies across time horizons, and the stringent requirements of real-time online prediction. These limitations underscore the need for more robust and adaptable modeling frameworks.

The IoV integrates vehicles, infrastructure, and cloud services, where trajectory prediction must address the variability and uncertainty of driving behaviors [2]. Among these challenges, colored noise—characterized by time-varying statistical dependencies—makes pattern recognition more difficult, especially when the noise exhibits temporal correlations (e.g., pink or brown noise). Additionally, GPS data are inherently sequential and reflect long-term dependencies due to road structure, traffic flow, and driver intent. Meeting the real-time requirements of IoV further necessitates models that are both efficient and resilient to fluctuating data quality.

Classical tracking methods, such as the Kalman filter [3], assume white Gaussian noise and rely on recursive estimation. These assumptions, however, break down in the presence of temporally correlated colored noise. In particular, temporal correlation in colored noise causes estimation bias to accumulate across time steps, which may eventually lead to divergence in the state estimates. For instance, when the measurement errors are correlated rather than independent, the filter repeatedly reinforces the same erroneous information instead of averaging it out. In recent years, deep learning approaches like Long Short-Term Memory (LSTM) and Gated Recurrent Units (GRUs) [4,5,6] have achieved success in modeling sequential dependencies. However, these deterministic models typically operate with fixed architectures and parameter sets, which limits their adaptability to dynamic and noisy environments. Moreover, their reliance on long-term dependencies makes them prone to instability in multi-step predictions, where small errors propagate and amplify across horizons, degrading overall robustness. To overcome these shortcomings, several works have explored more flexible sequence models with attention mechanisms, such as the Ada-STGMAT network [7], which adaptively handles spatiotemporal data in smart city environments.

Stochastic configuration networks (SCNs) [8] provide an alternative solution, incrementally constructing neural networks through a supervisory mechanism that adaptively regulates random parameter assignment. This design guarantees universal approximation and competitive generalization, while eliminating the need for backpropagation. Although SCNs were originally developed for general-purpose learning tasks, their architecture is particularly well-suited to scenarios involving nonstationary and noisy data due to their fast convergence and incremental learning strategy.

To address these limitations, we propose a probabilistic trajectory prediction framework that marries deterministic attention with stochastic learning via two ingredients: (1) a Bayesian linear head atop SCNs features that yields closed-form Gaussian predictive distributions and captures aleatoric uncertainty and (2) an ensemble of independently constructed SCNs configurations, each refitted with the Bayesian head, whose variability quantifies epistemic uncertainty. We combine these with a trainable attention encoder for context-aware feature extraction. The SCNs architecture is incrementally constructed under a supervisory criterion and then fixed; randomness in the constructive phase is used solely for ensemble-based uncertainty estimation and is not treated as posterior sampling over SCNs parameters. This hybrid attention-guided SCNs (AttSCNs) provides calibrated prediction intervals and context-sensitive processing for IoV trajectories while remaining lightweight at inference.

The structure of this paper is as follows: Section 2 introduces the aims and key contributions of this work. Section 3 presents a concise review of traditional and deep learning-based tracking methods, highlighting their strengths and limitations. Section 4 describes the architecture of the proposed AttSCNs framework, including details of the SCNs construction process and attention mechanism. Section 5 outlines the experimental setup and evaluation indicators and explains the Bayesian hyperparameter optimization strategy. Section 6 reports extensive experimental results and comparative analyses against recent baselines. Finally, Section 7 and Section 8 conclude this paper and outline future directions.

## 2. Aims and Contributions

The aims of this paper are twofold: (1) to enhance the robustness and probabilistic interpretability of SCNs for trajectory prediction under complex, colored-noise conditions and (2) to integrate SCNs with attention mechanisms to better capture long-range spatiotemporal dependencies in intelligent transportation scenarios. Based on these aims, our key contributions are as follows:

1. Explicit probabilistic AttSCNs. We provide an explicit probabilistic treatment for SCNs-based trajectory prediction by placing a Bayesian linear head on SCNs features and ensembling stochastic SCNs configurations. This yields closed-form Gaussian predictive distributions (aleatoric) and an epistemic component from configuration variability, enabling calibrated prediction intervals and uncertainty decomposition.
2. Attention-guided SCNs for robust sequence modeling. We propose AttSCNs, coupling a trainable attention encoder with an incrementally constructed SCNs layer. The attention module enhances long-range dependency learning, while the SCNs’ supervisory configuration improves robustness to colored noise and facilitates fast, non-iterative construction.
3. Uncertainty-aware evaluation and fair tuning. We employ strictly proper scoring rules and interval metrics (NLL, CRPS, PICP, and MPIW) alongside RMSE/MAE/SMAPE/$R^{2}$, with trajectory-level splits and multi-seed reporting. Extensive experiments demonstrate improved accuracy and calibration over strong baselines, while retaining real-time efficiency.

## 3. Related Work

Accurate long-term trajectory prediction is pivotal for intelligent transportation systems, as it underpins effective traffic flow management, enhances road safety, and facilitates optimal resource allocation [9]. Moreover, it serves as a cornerstone for the decision-making and path-planning modules of autonomous vehicles. However, in urban environments, GPS signals are frequently corrupted by complex colored noise—characterized by temporal correlations and non-uniform variance—which severely impairs the reliability of conventional prediction algorithms. These challenges highlight the pressing need for adaptive and robust modeling approaches that can effectively mitigate noise effects and maintain stable performance over extended forecasting horizons.

In the domain of GPS trajectory prediction, traditional methods primarily stem from tracking theory. The Kalman filter, introduced by Rudolf Emil Kalman, has long served as the cornerstone for such approaches [3]. It operates on Bayesian estimation principles, recursively computing optimal state estimates through prediction and update steps. These steps rely heavily on predefined motion and observation models to describe system dynamics and measurement relations, respectively. Variants of the Kalman filter, including the Constant Velocity (CV) [10], Constant Acceleration (CA) [11], Singer [12], and Current Statistical models [13], define target maneuvering using specific assumptions about the stochastic nature of acceleration. For instance, while the CV model assumes zero-mean white Gaussian noise in velocity, the Singer model introduces exponentially correlated stochastic processes, and the Current Statistical model adopts a Rayleigh distribution. Yet, the effectiveness of these filters depends significantly on the validity of such noise distribution assumptions, which rarely hold under the complex dynamics of urban transportation systems.

Measurement modeling further complicates Kalman-based tracking. Within the Internet of Vehicles (IoV), GPS sensors are widely used for position and velocity acquisition. However, their signals are frequently contaminated by colored noise, particularly pink noise [14], which invalidates the white Gaussian noise assumptions inherent in traditional Kalman filters. Such a mismatch leads to decreased estimation accuracy or even filter divergence. Although researchers have proposed adaptive Kalman filters to mitigate model uncertainty, such as by adjusting dynamic noise parameters or online estimation of process noise covariance, the fundamental limitations of model-based estimation frameworks constrain their effectiveness [15].

Ultimately, traditional methods exhibit significant shortcomings in addressing long-term prediction tasks. Rooted in process models with limited rank and rigid assumptions, these approaches struggle to capture the intricate temporal dependencies embedded in real-world GPS data. Consequently, their predictions often diverge over extended horizons, thereby reinforcing the need to shift toward data-driven alternatives that can better model complex spatiotemporal dynamics.

Deep neural networks (DNNs) represent a widely adopted data-driven approach that circumvents rigid model assumptions by learning complex input–output mappings from data. When trained on large-scale IoV datasets, DNNs have shown potential in predicting dynamic target attributes such as position, velocity, and acceleration. Notably, Bai et al. [16] proposed a recurrent auto-regressive neural framework for GPS/INS fusion. Similar advancements include the work of Aissa [17], who extended Kalman filters with neural architectures for robot trajectory tracking, and Markos [18], who applied Bayesian deep learning for unsupervised GPS trajectory segmentation.

Deep networks such as CNNs, LSTMs, and Transformers have further demonstrated their efficacy in capturing spatiotemporal dependencies. For example, Nawaz [19] combined CNNs and LSTMs for transportation mode classification using GPS and weather data. LSTM and GRU models effectively capture long-term temporal patterns [20,21,22], while hybrid models such as CNN-LSTM [23] extract both spatial and temporal features. The Transformer model [24,25] and its derivatives—Informer [26], Autoformer [27], and others—introduce self-attention mechanisms for long-range sequence modeling.

Additionally, probabilistic generative models such as Planar Flow-based VAEs (PFVAE) offer a promising avenue for learning complex data distributions [28]. Variational Bayesian En-Decoder models [29] have also demonstrated success in traffic flow prediction by explicitly modeling uncertainty and dynamic latent structures.

Despite their advancements, deep learning methods face persistent challenges in GPS trajectory prediction. First, the learning capacity of supervised networks is hindered by the nonstationary colored noise embedded in GPS signals. This often results in increasingly deep and complex models that require substantial data, training time, and computational resources to converge. In contrast, probabilistic neural architectures offer better generalization through explicit modeling of data uncertainty. Second, effective long-term prediction necessitates capturing extended temporal dependencies—a task that benefits from attention mechanisms [30] capable of highlighting relevant sequence features over time.

To address these limitations, our study proposes an attention-guided stochastic configuration network (AttSCNs) framework that integrates attention mechanisms with adaptive SCNs. SCNs are a class of randomized neural networks that incrementally construct their structure by dynamically configuring hidden nodes based on the residual error and supervisory criteria. This allows SCNs to flexibly adapt to complex, nonlinear, and multi-modal distributions, making them particularly suitable for modeling colored noise in GPS signals.

SCNs have demonstrated strong applicability in diverse fields, including finance [31], healthcare [32], structural reliability analysis [33], and traffic forecasting [34]. For instance, Y. Lin [34] applied SCNs for long-term traffic prediction in smart cities, showcasing their robustness and accuracy. In structural engineering, Li et al. [33] leveraged SCNs to predict structural reliability under high-dimensional nonlinear conditions. These applications underscore the efficacy of SCNs in capturing intricate temporal and statistical relationships.

Recent developments in SCNs methodologies have further expanded their capabilities. Wang and Felicetti [35,36] introduced Stochastic Configuration Machines (SCMs) and implemented FPGA-based architectures to enhance SCNs computation for real-time systems. Yan [37] combined Variational Mode Decomposition with SCNs for hydropower vibration prediction. In contrast, Li et al. [38] developed an online self-learning SCNs model for real-time stream classification in industrial contexts. Moreover, variants such as robust SCNs (RSCNs) [39], chaotic sparrow search algorithm SCNs (CSSA-SCNs) [40], and parallel SCNs (PSCNs) [41] have emerged to improve robustness, parameter tuning, and scalability.

Drawing inspiration from CSSA-SCNs and PSCNs, our proposed AttSCNs model incorporates Bayesian optimization to fine-tune key hyperparameters. This strategy enhances computational efficiency and prediction robustness. By combining attention-driven feature extraction with SCNs’ incremental learning capability, AttSCNs are well-equipped to handle long sequences, temporal noise, and complex data dynamics. Consequently, AttSCNs represent a powerful and efficient solution for trajectory prediction in IoV systems, where conventional neural and filter-based models struggle.

## 4. Materials and Methods

### 4.1. Architecture of AttSCNs

**Figure 1:**
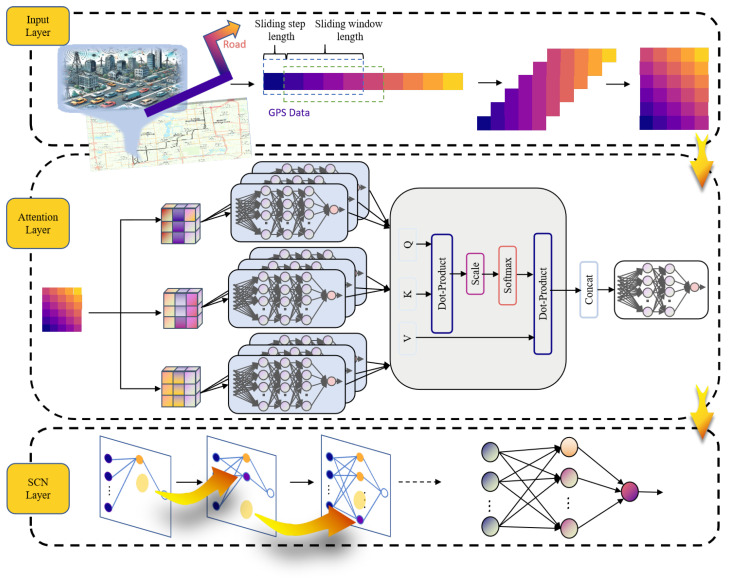
Model architecture of attention-guided stochastic configuration networks (AttSCNs).

The architecture of the proposed attention-guided stochastic configuration network (AttSCNs) model is illustrated in Figure 1. In this framework, GPS trajectories are first segmented into overlapping subsequences through a sliding-window strategy to generate input samples. The input layer receives raw multivariate time series data, which are often high-dimensional and contaminated by noise. An explicit probabilistic treatment is achieved by first placing a Bayesian linear head on the stochastic configurations of SCNs’ feature ensembles. An attention encoder is then applied to extract salient temporal features by projecting the inputs into queries (Q), keys (K), and values (V), computing scaled dot-product attention, and aggregating outputs from multiple attention heads. This process enables the model to selectively focus on time-relevant segments while suppressing irrelevant patterns, thereby enhancing feature representation.

The refined features are subsequently fed into the SCNs layer, which incrementally configures hidden nodes under a supervisory mechanism. Unlike conventional neural networks that rely on gradient-based optimization, SCNs construct their architecture in a non-iterative manner by selecting random parameters that satisfy a predefined consistency criterion. This approach enhances robustness and significantly reduces computational cost, particularly under colored-noise conditions common in GPS data. Although the SCNs architecture becomes fixed once the residual error falls below a threshold, the incremental construction process prior to convergence enables the network to flexibly approximate complex nonlinear dynamics.

To ensure convergence and prevent overfitting, AttSCNs employ three stopping criteria during SCNs construction: (1) the residual error norm falls below a predefined threshold ε; (2) the number of hidden nodes reaches a maximum value $L_{\max}$; or (3) the number of consecutive failed node configurations exceeds a preset limit. These conditions jointly ensure compactness and reliability of the model.

The AttSCNs model comprises three main modules: an input layer for raw data ingestion, an attention encoder for extracting dynamic temporal features, and a stochastic configuration layer for nonlinear modeling. This hybrid structure combines the strengths of attention and SCNs, enabling AttSCNs to handle long sequences, colored noise, and dynamic patterns with high robustness and accuracy.

### 4.2. Bayesian Linear Head for Probabilistic Treatment

The stochastic configuration networks (SCNs) generate an ensemble of hidden features $H=\left[h_{1},\ldots ,h_{L}\right]\in R^{N\times L}$ through randomized node construction. To enable explicit probabilistic modeling, we place a Bayesian linear head on top of *H*, treating the output weights β as random variables with a Gaussian prior:

(1) $$\beta ∼N(0,\gamma^{-1}I_{L}),\;\gamma >0,$$

where $I_{L}$ is the identity matrix and γ controls the prior’s precision. Given the observed targets $Y\in R^{N\times q}$, the likelihood is modeled as follows:

(2) $$Y=H\beta +\varepsilon ,\;\varepsilon ∼N(0,\sigma^{2}I_{N}),$$

with $\sigma^{2}$ as observation noise. The posterior distribution of β then follows from Bayes’ theorem:

(3) p(β|H,Y)∝p(Y|H,β)p(β).

Substituting Equations (1) and (2), the log-posterior becomes

(4) $$\log p\left(\beta |H,Y\right)=-\frac{1}{2\sigma^{2}}{∥Y-H\beta ∥}_{F}^{2}-\frac{\gamma }{2}{∥\beta ∥}_{F}^{2}+\mathrm{const},$$

which is maximized by the MAP estimator equivalent to ridge regression:

(5) $${\hat{\beta }}_{\mathrm{MAP}}={\left(H^{⊤}H+\lambda I_{L}\right)}^{-1}H^{⊤}Y,\;\lambda =\sigma^{2}\gamma .$$

The predictive distribution for a new input $x_{*}$ with feature $h_{*}=g\left(w^{⊤}x_{*}+b\right)$ is

(6) $$p\left(y_{*}|x_{*},H,Y\right)=N\left(h_{*}^{⊤}{\hat{\beta }}_{\mathrm{MAP}},\;h_{*}^{⊤}\Sigma h_{*}+\sigma^{2}\right),$$

where $\Sigma =\sigma^{2}{\left(H^{⊤}H+\lambda I_{L}\right)}^{-1}$ is the posterior covariance. This Bayesian treatment provides uncertainty quantification by propagating both feature randomness (from SCNs’ stochastic configurations) and weight uncertainty (from the linear head).

### 4.3. SCNs with Attention Encoder

Calculating the weights of each element in the input sequence is the fundamental concept of the attention mechanism (i.e., attention weights) to determine which parts are most important for the current task [42]. These weights are used to scale the input data, enabling the model to focus more effectively on key information. The attention weights create a weight matrix across the time dimension, with the sum of all weights in each row equal to 1. By executing multiple scaled dot-product calculations, the model can parallel extract multiple pieces of feature information from the data and pay different attention to various features, further enhancing its feature extraction capability and enabling it to capture more relevant information.

Given the input dataset $X=\{x_{1},x_{2},\ldots ,x_{N}\}$, where $x_{i}=\left\{x_{i,1},x_{i,2},\ldots ,x_{i,d}\right\}\in R$, assume the output $\mathrm{out}\in R^{t\times m}$ and $Q=K=V\in R^{t\times m}$, where *h* is the number of heads. Performing *h* linear transformations on Q, K, V separately, the *i*-th head’s output $Q_{i}$, $K_{i}$, $V_{i}\in R^{t\times d}$ is obtained as follows:

$$\begin{array}{cccc} q_{i} & =Qw_{q}^{i}, & i & =1,2,\ldots ,h\\ k_{i} & =Kw_{k}^{i}, & i & =1,2,\ldots ,h\\ v_{i} & =Vw_{v}^{i}, & i & =1,2,\ldots ,h \end{array}$$

(7) $$q_{i}\in R^{t\times d},\;k_{i}\in R^{t\times d},\;v_{i}\in R^{t\times d},\;d=\frac{m}{h}.$$

(8) $${\mathrm{AttentionWeight}}_{i}=\mathrm{softmax}\left(\frac{Q_{i}K_{i}^{T}}{\sqrt{d}}\right)\in R^{t\times t}.$$

(9) $${\mathrm{head}}_{i}={\mathrm{AttentionWeight}}_{i}V_{i}\in R^{t\times d},\;i\in \left[1\ldots h\right],\;\mathrm{out}=w\left[{\mathrm{head}}_{1};\ldots ;{\mathrm{head}}_{h}\right]\in R^{t\times m}.$$

As a randomized learning of neural networks, SCNs assign hidden nodes’ input weights and biases at random under a supervisory mechanism that adapts the sampling range, constructing the network incrementally. Beginning with a basic structure, hidden nodes are added until a probabilistic consistency criterion is satisfied.

Configuration of Hidden Layer Node: For a candidate node $h_{L}$ with parameters $\left(w_{L},b_{L}\right)$,

(10) $$h_{L}=g_{L}\left(w_{L}^{⊤}x+b_{L}\right).$$

For L−1 accepted nodes, the network output is

(11) $$f_{L-1}\left(X\right)=\sum_{j=1}^{L-1}\beta_{j}g_{j}\left(w_{j}^{⊤}X+b_{j}\right),\;L=1,2,\ldots ,L_{\max},$$

with residual

(12) $$e_{L-1}=Y-f_{L-1}\left(X\right).$$

A candidate is accepted when the supervisory score

(13) $$\xi_{L,q}=\frac{{\left(e_{L-1,q}^{⊤}h_{L}\right)}^{2}}{h_{L}^{⊤}h_{L}}-\left(1-r-\mu_{L}\right){∥e_{L-1,q}∥}^{2}$$

is non-negative for all outputs *q*, with r∼U(0,1) and $\mu_{L}\;↓\;0$. Summing over *q* yields $\xi_{L}=\sum_{q}\xi_{L,q}\geq 0$.

Output weights and maximum a posteriori (MAP) view. Let *H* stack all accepted hidden responses. Under a Gaussian prior $\beta \;∼\;N(0,\gamma^{-1}I)$ and noise y=Hβ+ε, $\varepsilon \;∼\;N(0,\sigma^{2}I)$, the MAP estimator equals ridge regression:

(14) $${\hat{\beta }}_{\mathrm{MAP}}=\arg\min_{\beta }{∥H\beta -Y∥}_{F}^{2}+\frac{\sigma^{2}}{\gamma }{∥\beta ∥}_{F}^{2}.$$

The deterministic SCNs prediction uses the mean $f=H\;{\hat{\beta }}_{\mathrm{MAP}}$, while our probabilistic AttSCNs further propagate posterior uncertainty as below.

### 4.4. Stochastic Configuration Networks from a Variational Perspective

The stochastic configuration process in SCNs can be interpreted through the lens of variational inference (VI), where the random weights and biases (w,b) play a role analogous to latent variables in a probabilistic model. Unlike traditional VI, which explicitly defines a variational posterior $q_{\phi }\left(\beta \right)$ over model parameters and optimizes it via the evidence lower bound (ELBO), SCNs induce randomness through stochastic sampling under supervisory constraints, effectively approximating an implicit variational distribution over network configurations.

In VI, latent variables (e.g., weights β) are treated as random quantities with a learned approximate posterior $q_{\phi }\left(\beta \right)$. In SCNs, the hidden layer parameters (w,b) are randomly sampled from a distribution (e.g., uniform or Gaussian) but are constrained by the supervisory mechanism, ensuring that each configuration contributes meaningfully to the ensemble. This process can be viewed as follows:

(15) (w,b)∼p(w,b|D)

where p(w,b|D) is an implicit posterior induced by the supervisory criteria. Unlike VI, which explicitly parameterizes $q_{\phi }\left(\beta \right)$ (e.g., as a Gaussian), SCNs do not learn a parametric posterior but instead rely on Monte Carlo sampling from an implicit distribution.

The supervisory mechanism in SCNs ensures that each random configuration improves the ensemble’s performance, analogous to how VI optimizes the ELBO to approximate the true posterior. The key difference is that SCNs do not perform gradient-based optimization over the latent space but instead enforce constraints on the stochastic weights:

(16) $$E_{\left(w,b\right)}\left[∥e_{L-1}-\xi_{L}g_{L}{∥}^{2}\right]\leq \eta_{L},$$

where $\eta_{L}$ controls the contribution of each random configuration. This resembles a constraint-based variational objective, where randomness is regulated rather than explicitly optimized.

While VI quantifies uncertainty through the spread of the learned posterior $q_{\phi }\left(\beta \right)$, SCNs estimate uncertainty empirically via ensemble variance:

(17) $${\mathrm{Var}}_{m}\left(\mu_{*}^{\left(m\right)}\right)\approx E_{\left(w,b\right)}\left[{\left(\mu_{*}\left(x_{*}\right)-{\bar{\mu }}_{*}\left(x_{*}\right)\right)}^{2}\right],$$

where $\mu_{*}^{\left(m\right)}$ is the prediction from the *m*-th stochastic configuration. This ensemble-based uncertainty is computationally efficient but lacks the theoretical guarantees of VI’s ELBO-optimized posterior.

The proposed AttSCNs have some key differences from traditional VI, including the following: Firstly, SCNs do not learn $q_{\phi }\left(\beta \right)$ but sample from an implicit distribution. Secondly, the proposed networks do not employ gradient-based ELBO optimization, in which supervisory constraints replace backpropagation through latent variables. Finally, SCNs avoid the memory cost of maintaining a full posterior over weights. This positions SCNs as a sampling-based alternative to VI, trading theoretical elegance for computational efficiency.

## 5. Experiment Strategy

### 5.1. Experiment Setup and Evaluation Indicators

All experiments are conducted on a high-performance computing server equipped with eight NVIDIA Tesla P40 GPUs, each operating at 1531 MHz with 24 GB of memory.

To comprehensively evaluate model performance, we adopt four widely used regression metrics: Root Mean Square Error (RMSE), Mean Absolute Error (MAE), Symmetric Mean Absolute Percentage Error (SMAPE), and Coefficient of Determination ($R^{2}$). Their mathematical definitions are as follows:

(18) $$\mathrm{RMSE}=\sqrt{\frac{1}{n}\sum_{i=1}^{n}{\left({\hat{y}}_{i}-y_{i}\right)}^{2}}$$

(19) $$\mathrm{MAE}=\frac{1}{n}\sum_{i=1}^{n}\left|{\hat{y}}_{i}-y_{i}\right|$$

(20) $$\mathrm{SMAPE}=\frac{100\%}{n}\sum_{i=1}^{n}\frac{|{\hat{y}}_{i}-y_{i}|}{\left(|{\hat{y}}_{i}\left|+\right|y_{i}|\right)/2}$$

(21) $$R^{2}=1-\frac{\sum_{i=1}^{n}{\left(y_{i}-{\hat{y}}_{i}\right)}^{2}}{\sum_{i=1}^{n}{\left(y_{i}-\bar{y}\right)}^{2}}$$

where *n* is the total number of test samples, $y_{i}$ is the *i*-th ground truth value, ${\hat{y}}_{i}$ is the corresponding predicted value, and $\bar{y}$ denotes the mean of the actual values. For RMSE, MAE, and SMAPE, lower values indicate higher prediction accuracy. In contrast, higher $R^{2}$ values signify better model fit and explanatory power.

### 5.2. Uncertainty Metrics and Evaluation Protocol

We complement RMSE/MAE/SMAPE/$R^{2}$ with uncertainty-centric metrics. In our framework, probabilistic predictions arise explicitly from a Bayesian linear head placed on top of the SCNs features. Given the hidden design matrix *H*, we place a Gaussian prior on the output weights and obtain closed-form Gaussian predictive distributions for each target, with means $H_{*}\mu_{\beta }$ and isotropic multi-output covariance $\left(H_{*}\Sigma_{\beta }H_{*}^{⊤}+\sigma^{2}\right)\;I_{m}$ (aleatoric component). In addition, by re-drawing the stochastic SCNs configurations *M* times and refitting the Bayesian head, we quantify epistemic uncertainty from the variability of predictive means across configurations; the sum of aleatoric and epistemic parts yields the total predictive uncertainty used to form prediction intervals.

The following metrics, therefore, evaluate the quality and calibration of our prediction distributions.

(i) Negative Log-Likelihood (NLL). For a Gaussian predictive density $N(\mu_{i},\sigma_{i}^{2})$ at target $y_{i}$,
  (22) $$\mathrm{NLL}=\frac{1}{n}\sum_{i=1}^{n}\left[\frac{{\left(y_{i}-\mu_{i}\right)}^{2}}{2\sigma_{i}^{2}}+\frac{1}{2}\log\left(2\pi \sigma_{i}^{2}\right)\right].$$
  For multi-output trajectories, we compute the NLL/CRPS per scalar target under the isotropic predictive covariance assumption above and report the average across dimensions and time steps.
(ii) Continuous Ranked Probability Score (CRPS). For Gaussian forecasts, we use the closed-form CRPS; the lower the value, the better.
(iii) Prediction Interval Coverage Probability (PICP) and Mean Prediction Interval Width (MPIW). For (1−α) intervals,
  (23) $$\mathrm{PICP}=\frac{1}{n}\sum_{i=1}^{n}1\left\{y_{i}\in {\mathrm{PI}}_{1-\alpha }\left(x_{i}\right)\right\},\;\mathrm{MPIW}=\frac{1}{n}\sum_{i=1}^{n}\left[U_{i}-L_{i}\right].$$
  Well-calibrated models have PICP≈1−α with short MPIW.

We adopt the following evaluation protocol: (i) split at trajectory level to prevent leakage; (ii) repeat each experiment with five random seeds, reporting mean ± std; (iii) for AttSCNs, set M=10 unless otherwise noted; (iv) tune baselines fairly; and (v) report NLL/CRPS/PICP/MPIW alongside RMSE/MAE/SMAPE/$R^{2}$. All numerical uncertainty results are reported in Section 6 (see Table 1).

### 5.3. Bayesian Hyperparameter Optimization

To further enhance model performance, we apply Bayesian optimization to tune critical hyperparameters of AttSCNs. The hyperparameter search space is defined in Table 2. The optimization explores this space using a Gaussian process surrogate and the Expected Improvement (EI) acquisition function. For reproducibility, we ran a capped budget of 70 trials per dataset/horizon (10 random warm-up evaluations + 60 BO iterations). Each trial trained the candidate configuration for up to 300 epochs with early stopping (patience = 20) and was evaluated by the validation objective (NLL + CRPS + 0.1 × RMSE). A wall-clock budget of 1 GPU-hour on an NVIDIA Tesla P40 was enforced for each BO run; in practice, the iteration cap was typically reached before the time limit was reached.

After iterative exploration, the optimal configuration was identified as follows:

- $L_{\max}$ (Maximum number of hidden neurons): 300;
- ϵ (Probabilistic Constraints): 0.0001;
- $T_{\max}$ (Maximum random configurations): 200;
- Lambdas (Range of random weights): [0.001, 0.005, 0.008, 0.01, 0.1, 0.5, 1, 5, 10, 30, 50, 100, 150, 200, 250];
- *r* (Regularization parameter): [0.9, 0.99, 0.999, 0.9999, 0.99999].

## 6. Experiment Results

### 6.1. Beijing Vehicle GPS Trajectory Dataset

We used the Geolife Beijing vehicle GPS trajectory dataset (https://www.microsoft.com/en-us/research/publication/t-drive-trajectory-data-sample/ accessed on 30 July 2025), which is a subset of the T-Drive trajectory dataset. This dataset comprises one-week trajectories of 10,357 taxis, totaling about 15 million data points and covering a total distance of approximately 9 million kilometers. The dataset features various sampling intervals, without missing data points, across different trajectory groups.

**Figure 2:**
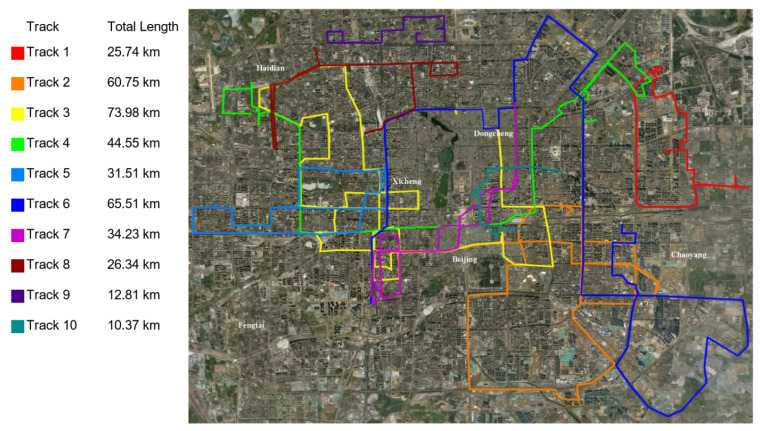
Ten example trajectories from the Geolife Beijing vehicle GPS motion track dataset.

From this comprehensive dataset, we selected one representative group of trajectories, containing 13,987 GPS coordinates, which was used for training and evaluation. Of these, 80% were used as the training set for the model, while the remaining 20% comprised the test set. Figure 2 shows ten example trajectories from this dataset.

### 6.2. Compared with State of the Art

**Figure 3:**
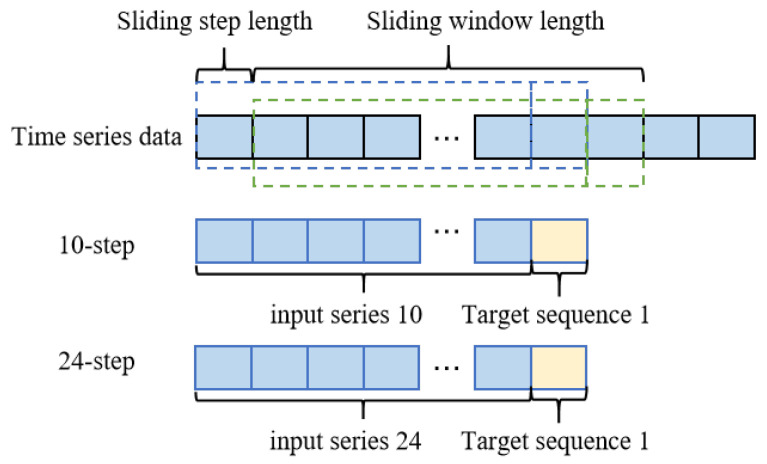
Sliding window for 10-step and 24-step predictions.

As shown in Figure 3, suppose that the data $X={\left[x_{1},x_{2},\ldots ,x_{m}\right]}^{T}$ is time series data with a length of *m*. The input length *t* for the prediction model is 10 or 24, and the prediction length τ is 1. The data is divided into a length window t+τ for supervised learning, with a sliding step length *s* of 1. After the first operation of the sliding window, the first sequence $X^{1}$ is obtained as follows:

(24) $$X^{1}={\left[x_{1},x_{2},\ldots ,x_{t+\tau }\right]}^{T}$$

The second sequence $X^{2}$ is

(25) $$X^{2}={\left[x_{s+1},x_{s+2},\ldots ,x_{s+t+\tau }\right]}^{T}$$

The *i*-th sequence $X^{i}$ is

(26) $$X^{i}={\left[x_{s*(i-1)+1},\ldots ,x_{s*(i-1)+t+\tau }\right]}^{T}$$

Inputs for the model are made from the first *t* data points in each group, and the last τ data points are employed as the target series. Figure 3 displays the input length *t* as 50 and the prediction lengths τ as 10 or 24.

We compared the proposed AttSCNs model with the following three groups:

Group 1: Comparing with the classical Kalman filter using a variety of process models, such as CA [11], CV [10], Singer [12], and Current Statistical [13]. These methods serve as historical baselines to show the gap with modern approaches, while the main comparison focuses on Group 2 and Group 3 models that better highlight the contributions of AttSCNs.

Group 2: Comparing with the deep learning networks, such as GRU [20], LSTM [21], CNN-LSTM [23], Transformer [24], Informer [26], and Autoformer [27], and PFVAE [28].

Group 3: Comparing with the base SCNs [8].

The experimental parameter configurations included GRU, LSTM, CNN-LSTM, Transformer, Informer, Autoformer, PFVAE, SCNs, and AttSCNs, which were analyzed for predictive accuracy employing a sliding-window framework of 50 steps predicting 10 steps and 50 steps predicting 24 steps. Before applying the sliding-window operation, the trajectories were first split into training and testing sets at the trajectory level, ensuring that no overlapping windows appeared across the two sets and thereby avoiding potential data leakage. Each model’s input sequence length is standardized at 50 steps, with output sequence lengths set at 10 or 24 steps.

For baseline models, the initial default settings were a learning rate of 0.0001, 100 training epochs, and a batch size of 32. These settings resulted in suboptimal performance. We therefore performed hyperparameter tuning: for Transformer-based models (Transformer, Informer, and Autoformer), we conducted a grid search on the validation set, exploring learning rates (0.0005–0.005), batch sizes (64–128), and dropout rates (0.1–0.3). The final tuned configuration adopted for fair comparison was a learning rate of 0.001, 300 epochs, and a batch size of 100, as reported in Table 3. Although extending training to 1000 epochs could further improve results, the computational time was prohibitively long, so we selected the current tuned settings as a balanced trade-off between accuracy and efficiency. This ensures that all baseline models were fairly optimized and not disadvantaged by under-tuning.

Two cases are included:

Case 1: The proposed AttSCNs model will predict the next 10 steps based on Beijing vehicle GPS trajectory data. The prediction results will be derived from the existing model and algorithm, providing an assessment of future trends.

Case 2: The proposed AttSCNs model will be used to predict the next 24 steps. This involves a more extended time range and requires the model to be more capable of capturing long-term dependencies in the time series data, which will improve the accuracy and reliability of the predictions.

### 6.3. Uncertainty and Calibration

We evaluate probabilistic performance using the metrics defined in Section 5.2. Table 1 reports results for 10-step predictions.

As detailed in Section 4.4, AttSCNs yield explicit Gaussian predictive distributions by placing a Bayesian linear head on SCNs features, with predictive mean $H_{*}\mu_{\beta }$ and isotropic multi-output covariance $\left(H_{*}\Sigma_{\beta }H_{*}^{⊤}+\sigma^{2}\right)\;I_{m}$ (aleatoric component). Epistemic uncertainty is captured by re-drawing the stochastic SCNs configurations *M* times and re-fitting the Bayesian head; the total predictive uncertainty is the sum of aleatoric and epistemic parts. Hence, the metrics in Table 1 assess the calibration and sharpness of full predictive distributions, not just point errors.

AttSCNs achieve the lowest NLL and CRPS and the narrowest 90% intervals while maintaining near-nominal coverage (PICP =0.89 with target 0.90), indicating well-calibrated yet sharp predictive densities. Relative to PFVAE, AttSCNs reduce CRPS by 40.6% (2.62 vs. 4.41) and MPIW by 40.5% (15.29 vs. 25.70), while also improving NLL by 5.7% (4.11 vs. 4.36). Compared with SCNs equipped with a ridge head, AttSCNs reduce CRPS/MPIW by about 41.8% and improve NLL by 6.4%, demonstrating that the Bayesian linear head (together with configuration ensembling) yields materially better-calibrated uncertainty than deterministic regression on the same features. By contrast, some deterministic sequence models show much wider intervals at lower coverage (e.g., Transformer: MPIW =128.0 with PICP =0.84), evidencing miscalibration.

To further strengthen the robustness of the conclusions, we performed formal statistical testing and effect size analysis on the results in Table 1. For each baseline versus AttSCNs, paired differences were formed and evaluated with a two-sided paired permutation test (10,000 sign flips) and with *p*-values adjusted across metrics and models using the Holm–Bonferroni procedure (α=0.05). We also report Cliff’s δ with 95% BCa bootstrap confidence intervals, as well as paired Cohen’s *d* and paired *t*-test statistics. The results show that AttSCNs achieve statistically significant improvements over all baselines in NLL and CRPS (adjusted p<0.01, at least moderate and often large effect sizes), and consistently narrower MPIW compared with all baselines (adjusted p<0.01). At the same time, differences in PICP relative to SCNs and PFVAE are not significant. Overall, these findings confirm that AttSCNs deliver sharper predictive intervals with sound calibration in a statistically significant manner.

### 6.4. Case 1: 10-Step Predictions

Table 4 presents the comprehensive results of the 10-step predictions for each model. The experimental protocol involved a comparative assessment of these models under noisy conditions. The effectiveness of each model was evaluated using several metrics, including RMSE, MAE, SMAPE, and $R^{2}$ value. To enhance reliability, all results are reported as mean ± standard deviation across five independent runs, thereby capturing performance variability. This detailed evaluation elucidates the capabilities and limitations of AttSCNs across various modeling scenarios, providing an extensive benchmark for comparison with traditional and pioneering machine learning techniques.

Table 4 shows that our proposed method has the lowest RMSE, MAE, and SMAPE, as well as the highest $R^{2}$. Specifically, our approach achieves an RMSE of 8.45, an MAE of 4.64, an SMAPE of 0.15, and an $R^{2}$ of 0.99981. Table 4 further reveals that traditional estimation methods rely heavily on parameter settings, which often require extensive expertise and are difficult to optimize. In contrast, our AttSCNs model outperforms the other 12 models evaluated in this study, as evidenced by a 98.61%, 96.88%, 97.20%, 96.52%, 58.70%, 52.53%, 65.50%, 84.80%, 87.26%, 82.03%, 35.10%, and 36.51% reduction in RMSE and a 99.06%, 97.95%, 98.13%, 97.79%, 70.91%, 68.48%, 78.70%, 89.37%, 91.05%, 87.32%, 55.21%, and 38.05% decrease in MAE for each of the comparison models. Additionally, our AttSCNs model decreases SMAPE by 98.65%, 97.97%, 98.15%, 97.84%, 67.39%, 64.29%, 76.56%, 97.90%, 98.42%, 97.60%, 57.14%, and 42.31%, respectively. The specific calculation formula is as follows:

(27) $$\begin{array}{c} \mathrm{Percentage}\;\mathrm{Improvement}=\left(\frac{\mathrm{Comparison}\;\mathrm{Model}’s\;\mathrm{Metric}-\mathrm{AttSCNs}’s\;\mathrm{Metric}}{\mathrm{Comparison}\;\mathrm{Model}’s\;\mathrm{Metric}}\right)\times 100\% \end{array}$$

In the 10-step prediction scenario, the performance of Group 1 models, including CA [11], CV [10], Singer [12], and Current Statistical [13], was notably poor, with $R^{2}$ values being very low or even negative. This indicates a significant discrepancy between the predicted trajectories and the reference values, suggesting that the predictions tend to diverge. The underlying reason for this divergence is that these classical models have process models that are not full-rank and possess unstable structures. As a result, they can make predictions only within a minimal horizon before requiring observational data for correction. In the context of 10-step forecasts, these models must iteratively compute forward ten times, making them prone to divergence.

**Figure 4:**
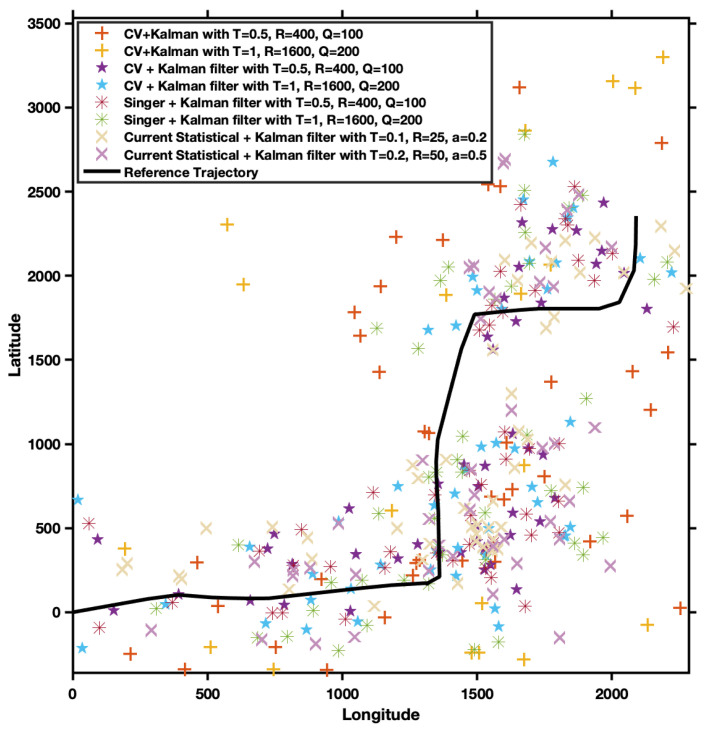
Comparison of GPS vehicle trajectories in Beijing estimated by Group 1 models.

Figure 4 presents the prediction trajectory results for Group 1 models. The predictions of classical estimation models deviate markedly from the reference values, leading to substantial errors. Additionally, we found that these classical estimation methods are susceptible to parameter selection, leading to considerably different prediction results with varying parameters. In particular, prediction using the CV model with parameters T = 1, R = 1600, and Q = 200 shows a divergent trend. Among the classical models, the CV model performs the worst, mainly due to its assumption of constant velocity for the target. When the target dynamics do not meet this assumption, the performance of the estimation and prediction deteriorates significantly. In contrast, the Singer model and the Current Statistical model demonstrate improved prediction performance, as these models assume more complex dynamics for target motion, aligning more closely with the actual situation. However, in general, the prediction results based on classical estimation methods are inadequate, which can be attributed to the complex dynamics of the target motion.

**Figure 5:**
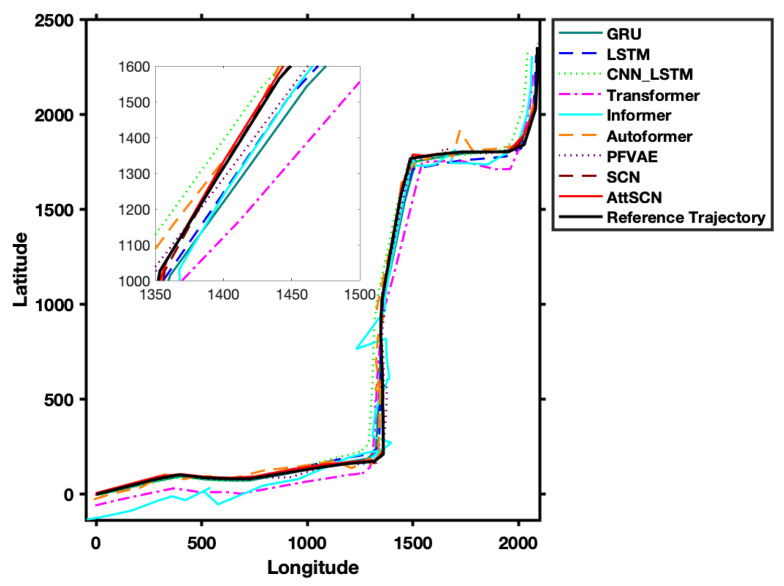
Comparison of GPS motion trajectories of vehicles in Beijing estimated by Group 2 and 3 models.

Compared to classical estimation models, the deep models employed in Groups 2 and 3, such as Transformer [24], Informer [26], Autoformer [27], PFVAE [28] (Planar Flow VAE), and SCNs [8], demonstrate significant advantages (shown in Figure 5). This improvement can be attributed to their ability to capture complex patterns and dynamics in the data, which classical models struggle with. Notably, the proposed AttSCNs model performs exceptionally well. For instance, when compared with the baseline SCNs, AttSCNs reduced RMSE from 13.31 to 8.45, lowered MAE from 7.49 to 4.64, and decreased SMAPE from 0.26 to 0.15, while improving $R^{2}$ from 0.99937 to 0.99981. These quantitative improvements provide clear evidence of the effectiveness of AttSCNs in achieving more stable and accurate trajectory predictions. This success can be attributed to their attention mechanism, which effectively focuses on relevant aspects of the input data, allowing for more accurate predictions by capturing temporal dependencies and variations in the target’s motion.

However, Informer performs relatively poorly. Its design, though optimized for efficiency and long sequences, may be less effective in capturing the specific dynamics of target motion. The model may overlook critical features necessary for accurate predictions, resulting in suboptimal results compared to other deep learning approaches.

**Figure 6:**
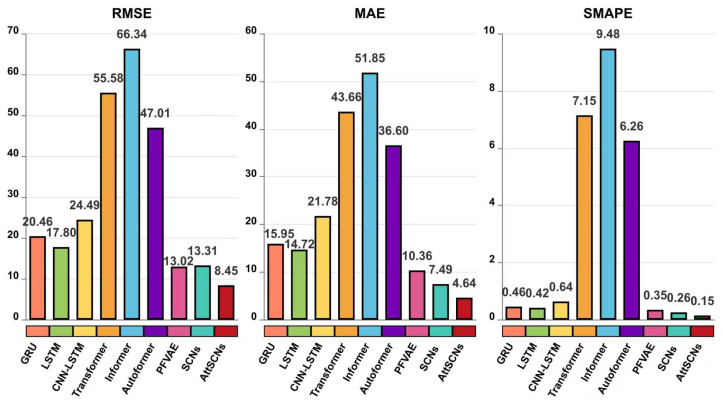
RMSE, MAE, and SMAPE comparison for 10-step predictions: AttSCNs vs. Group 2 and Group 3 models.

From Figure 6, it is evident that the proposed AttSCNs achieve the lowest values in terms of RMSE, MAE, and SMAPE. This indicates that AttSCNs have superior predictive accuracy compared to other models evaluated. On the other hand, the Informer model exhibits the most significant errors (just like those shown in Figure 5), which can be attributed to several factors. One possible reason is that the Informer model might not effectively capture the underlying temporal patterns or dependencies inherent in the dataset. Additionally, its architecture might not be as well-suited for the specific characteristics or noise present in the data, leading to suboptimal performance. Furthermore, the parameter tuning or optimization strategy utilized for the Informer model might not have been as rigorous or practical as that for AttSCNs, contributing to its relatively poor performance. Overall, these aspects suggest that while the Informer model is effective in many contexts, it may not be the best fit for the particular dataset or prediction task at hand, as demonstrated by its higher error rates.

### 6.5. Case 2: 24-Step Predictions

Similarly, Table 5 shows that the performance of Group 1 models—CA [11], CV [10], Singer [12], and Current Statistical [13]—was even poorer, with $R^{2}$ values almost negative. As previously analyzed, classical estimation methods for motion models and Kalman filters are unsuitable for long-term trajectory predictions. For modern deep learning models (Group 2 and Group 3), both mean performance and standard deviations are reported, ensuring fair comparison with AttSCNs. The proposed AttSCNs model obtained an RMSE improvement of 53.88%, 37.96%, 47.34%, 79.10%, 82.62%, 84.53%, 36.81%, and 22.67%, as well as an MAE improvement of 62.94%, 58.73%, 63.80%, 85.05%, 87.31%, 88.00%, 59.09%, and 41.67%, when compared to the performance of the GRU [20], LSTM [21], CNN-LSTM [23], Transformer [24], Informer [26], Autoformer [27], PFVAE [28], and SCNs [8] models, respectively. Moreover, SMAPE improved by 69.23%, 68.63%, 70.91%, 97.91%, 98.75%, 98.01%, 74.19%, and 61.90%, respectively. Furthermore, our AttSCNs model achieves the best $R^{2}$, implying an optimal fit between the estimated and observed values.

**Figure 7:**
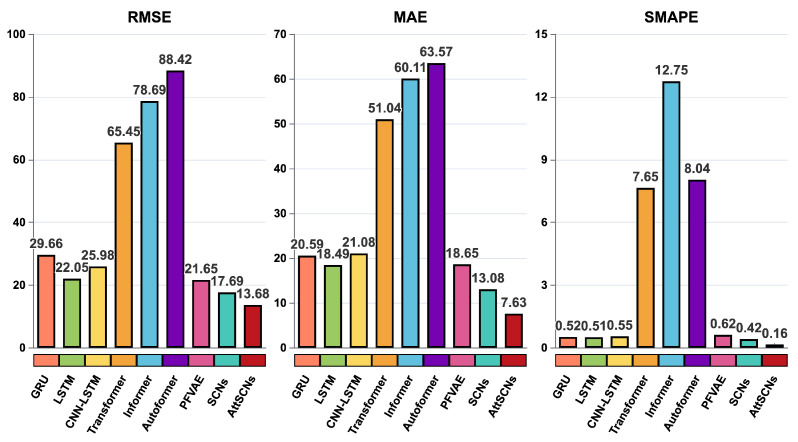
RMSE, MAE, and SMAPE comparison for 24-step predictions: AttSCNs vs. Group 2 and Group 3 models.

We can observe that the trends in Figure 7 are very similar to those in Figure 6. Our proposed AttSCNs model continues to achieve the fewest errors in terms of RMSE, MAE, and SMAPE; the performance of the Transformer, Informer, and Autoformer models declines significantly, with notable increases in RMSE, MAE, and SMAPE. The “former” series models struggle to capture the dynamics of maneuvering targets accurately. This may be due to a few reasons. Firstly, the architectures of these models might not be well-suited for capturing the complex and nonlinear temporal dependencies required for long-term predictions of dynamic systems. Additionally, the models may have insufficient mechanisms to handle varying velocities or abrupt changes in direction, which are common in maneuvering targets. Finally, the models may lack robustness against the increased uncertainty and noise that typically accompany longer prediction horizons, leading to larger errors in their predictions.

**Figure 8:**
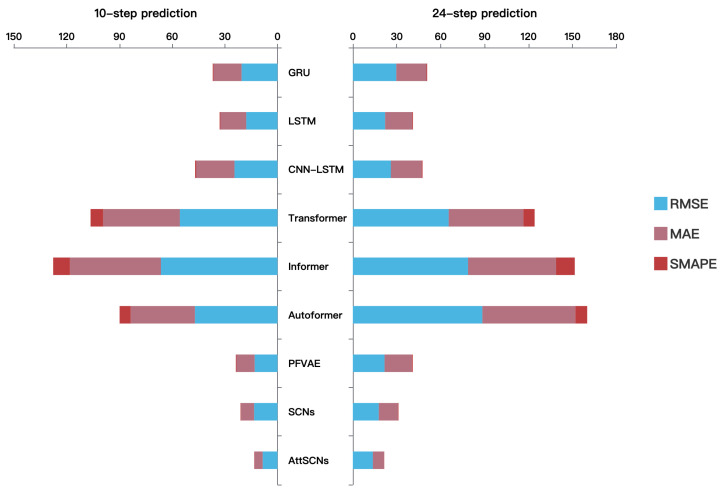
Comparison of 10-step and 24-step prediction performance across models.

In Figure 8, we present a stacked chart of RMSE, MAE, and SMAPE for two prediction lengths: 10-step and 24-step predictions. Unlike tables that emphasize exact values, this visualization highlights relative performance trends across multiple error metrics, thereby complementing the numerical results in Table 4 and Table 5 and enabling an intuitive, side-by-side comparison of different models. It can be seen that SMAPE is relatively large for the Informer series, while it is smaller for other models. Moreover, the proposed AttSCNs model achieves consistently lower values across all three error metrics and both horizons in the stacked chart, making its superiority in both short-term and long-term forecasting more intuitive. Therefore, the model proposed in this paper offers excellent error control capability, providing an effective method for long-term time series forecasting tasks.

**Figure 9:**
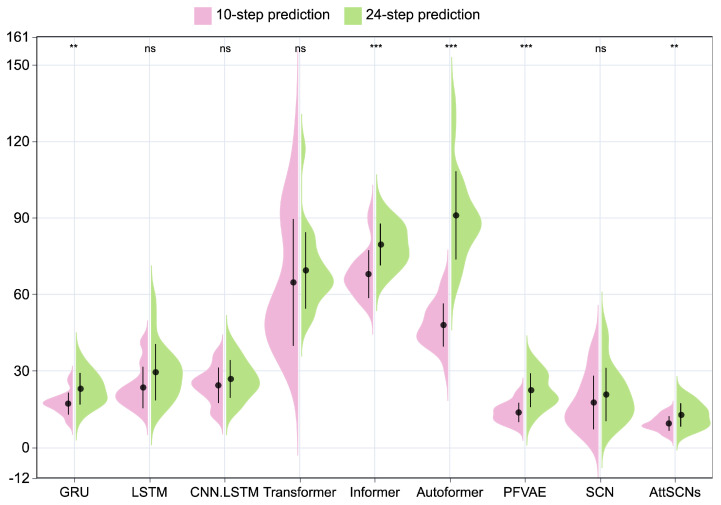
RMSE violin plot comparing models with 10-step and 24-step predictions from 20 independent repetitions on the Beijing vehicle GPS motion trajectory dataset. Significance codes: ** (p<0.01), *** (p<0.001), and ns (p≥0.05).

The violin plot presented in Figure 9 demonstrates the density and distribution characteristics of two prediction horizons, employing a *t*-test to assess statistical differences between the groups, with significance levels denoted by the markers * for *p* < 0.05, ** for *p* < 0.01, and *** for *p* < 0.001, and ns to indicate a lack of significance. RMSE was selected as the error metric in this visualization because it penalizes larger errors more heavily, thereby serving as a sensitive indicator of performance variability across repeated runs. While MAE and SMAPE are also valuable measures and are comprehensively reported in Table 4 and Table 5, RMSE provides the clearest visualization of distributional differences across models. The plot reveals that the proposed AttSCNs model exhibits a reduced range of error fluctuations, a more concentrated error distribution, and enhanced stability compared to baselines. Moreover, the predictive performance for the 24-step prediction is generally inferior to that of the 10-step prediction, as evidenced by elevated average errors and more dispersed distributions. Notably, the Informer series demonstrates robustness, with the error distribution for 24-step prediction closely resembling that of the 10-step case, reflecting the effectiveness of its sparse attention mechanism in capturing long-term dependencies.

### 6.6. Ablation Study

Table 6 reports the ablation results of AttSCNs, from which the contribution of each component can be explicitly observed. The SCNs-only variant achieves considerably lower errors than the attention-only model, confirming that SCNs provide the essential robustness for handling noisy GPS trajectories. When attention is integrated with SCNs (AttSCNs with default parameters), performance is further improved, demonstrating the benefit of attention in capturing long-term temporal dependencies.

To explicitly evaluate the contribution of Bayesian optimization, we compared the performance of AttSCNs before and after optimization. Without optimization, the AttSCNs model already outperforms conventional baselines; however, after Bayesian optimization, both prediction accuracy and robustness improved significantly, demonstrating the effectiveness of the hyperparameter search. This process systematically identified parameter values that achieved the best trade-off between predictive accuracy and model complexity. The direct comparison confirms that Bayesian optimization yields tangible performance gains, thereby justifying its inclusion in the trajectory prediction pipeline.

Overall, the ablation results indicate that SCNs constitute the backbone of the architecture, attention offers complementary gains, and Bayesian optimization provides additional refinement for optimal performance.

### 6.7. Discussion on the Real-Time Performance

To highlight the real-time advantages of the proposed AttSCNs model, this study emphasizes the significance of parameter count, which directly affects computational efficiency and memory consumption. The AttSCNs model contains only 126,252 parameters, a number close to that of GRU (121,400) and LSTM (161,600) and slightly smaller than that of CNN-LSTM (168,000). In contrast, Transformer, Informer, and Autoformer require 8,652,800, 8,652,800, and 3,293,696 parameters, respectively, which are one to two orders of magnitude higher than those of the other models. Despite the similarity in scale to GRU, LSTM, and CNN-LSTM, AttSCNs consistently achieve superior prediction accuracy.

Parameter count provides an intuitive measure of efficiency, but real-time capability is better characterized by computational complexity and inference latency. To this end, we analyzed multiply–accumulate operations (MACs) and estimated latency on representative edge hardware, including an ARM Cortex-A76 CPU (2.8 GHz) and an NVIDIA Jetson Xavier NX GPU (21 TOPS). The comparative results are summarized in Table 7.

As shown in Table 7, AttSCNs incur the lowest computational burden, requiring only ∼$6.1\times 10^{5}$ MACs per step, and achieve an estimated inference latency of 0.8–1.2 ms on CPU and less than 0.1 ms on GPU. These results demonstrate that AttSCNs not only reduce parameter count but also satisfy real-time latency requirements, making them highly suitable for deployment in vehicle trajectory prediction tasks on edge devices.

The superior prediction accuracy, combined with lightweight computational cost, ensures that AttSCNs can efficiently process high-throughput data streams under stringent latency constraints. The compact parameter scale leads to lower memory consumption and faster execution, thereby facilitating rapid model updates and stable real-time operation. Such efficiency is especially critical in Internet of Vehicles (IoV) applications, where timely processing of sequential data directly impacts navigation safety and decision-making.

Another distinctive strength of AttSCNs lies in the integration of attention mechanisms with stochastic configuration networks. This hybrid design enhances robustness against colored noise and improves the model’s ability to capture long-term dependencies in GPS signals. Compared with rigid architectures such as GRU and LSTM, AttSCNs can dynamically adapt to complex spatiotemporal patterns, ensuring both accuracy and resilience in noisy environments.

In summary, AttSCNs achieve a favorable balance of accuracy, efficiency, and robustness. Their educed complexity and real-time responsiveness underscore their practical value for IoV systems, where rapid and reliable trajectory prediction is essential.

## 7. Limitations and Future Work

This study employs trajectory data from a single city and domain, which may limit the generalizability of the findings; performance could vary under different traffic conditions, road network configurations, or GPS data quality. From a modeling perspective, the current Bayesian linear head adopts an isotropic multi-output covariance assumption for computational tractability. While this simplification proves effective, it may not fully capture the correlation structure of prediction errors across different dimensions or time horizons. Although our uncertainty evaluation incorporates multiple metrics (NLL, CRPS, PICP, and MPIW), it does not yet include comprehensive diagnostic tools such as reliability diagrams, expected calibration error (ECE), or probability integral transform (PIT) analysis. Additionally, while we report means and standard deviations across multiple random seeds, some experiments have relatively small sample sizes; more robust statistical inference (e.g., effect size estimation via bootstrap or permutation testing) would further strengthen the validity of our conclusions.

Future work will focus on two key directions: (i) conducting uncertainty decomposition studies using normalizing-flow techniques to better model multi-modal dynamics and non-Gaussian noise patterns and (ii) developing Bayesian-inspired neural architecture search methods to jointly optimize SCNs’ constructive policies and attention mechanisms (e.g., through evolutionary or Monte Carlo strategies), aiming to improve both accuracy and calibration metrics (CRPS and NLL). Our long-term objective is to develop a robust prediction system that maintains well-calibrated uncertainty estimates under domain shifts—particularly crucial for safety-critical applications in urban canyons and adverse weather conditions—while remaining computationally efficient for edge device deployment.

## 8. Conclusions

This paper presents AttSCNs, a novel hybrid framework for trajectory prediction that combines a trainable attention encoder with stochastic configuration networks (SCNs) and a Bayesian linear output layer. The attention mechanism learns context-aware temporal features, while SCNs are incrementally constructed under a supervisory criterion to provide lightweight, noise-resistant representations. The Bayesian linear head generates closed-form Gaussian predictive distributions, enabling principled quantification of aleatoric uncertainty. To model epistemic uncertainty, we employ an ensemble of independently constructed SCNs configurations, each equipped with a refitted Bayesian head; the variance across ensemble predictions is combined with the analytic aleatoric uncertainty to produce calibrated prediction intervals. Notably, Bayesian optimization is used exclusively for hyperparameter tuning (e.g., constructive limits, attention width, and ensemble size) against an uncertainty-aware validation objective—it does not perform posterior inference or directly generate uncertainty estimates.

AttSCNs demonstrate significant practical advantages: once constructed, the architecture remains fixed and requires minimal computational resources during inference, making it particularly suitable for real-time applications in the Internet of Vehicles (IoV) domain. Extensive evaluation on real-world GPS datasets shows that AttSCNs consistently outperform competitive baselines in prediction accuracy while delivering well-calibrated uncertainty estimates. These capabilities enable important downstream applications including risk-aware filtering, adaptive horizon control, and safety-margin decision making.

## Figures and Tables

**Table 1:** Uncertainty metrics on 10-step predictions (mean ± std over 5 runs).

Model	NLL	CRPS	PICP@90%	MPIW@90%
Transformer	5.60 ± 0.17	21.94 ± 1.54	0.84 ± 0.03	128.00 ± 7.68
GRU	4.69 ± 0.14	7.50 ± 0.53	0.86 ± 0.02	43.75 ± 2.63
LSTM	4.55 ± 0.14	6.53 ± 0.46	0.86 ± 0.02	38.07 ± 2.28
SCNs	4.39 ± 0.13	4.50 ± 0.32	0.88 ± 0.02	26.27 ± 1.58
PFVAE	4.36 ± 0.13	4.41 ± 0.31	0.88 ± 0.02	25.70 ± 1.54
AttSCNs	4.11 ± 0.12	2.62 ± 0.18	0.89 ± 0.01	15.29 ± 0.92

**Table 2:** Hyperparameter search space for Bayesian optimization.

Hyperparameter	Candidate Values
$L_{\max}$	50, 100, 200, 300
ϵ	0.00001, 0.0001, 0.001, 0.01
$T_{\max}$	50, 100, 150, 200, 250, 300
Lambdas	[0.01, 0.1, 0.5, 1, 5, 10, 30, 50, 100, 150, 200, 250], [0.008, 0.01, 0.1, 0.5, 1, 5, 10, 30, 50, 100, 150, 200, 250], [0.005, 0.008, 0.01, 0.1, 0.5, 1, 5, 10, 30, 50, 100, 150, 200, 250], [0.001, 0.005, 0.008, 0.01, 0.1, 0.5, 1, 5, 10, 30, 50, 100, 150, 200, 250], [0.001, 0.005, 0.008, 0.01, 0.1, 0.5, 1, 5, 10, 30, 50, 100, 200, 300, 500]
*r*	[0.9, 0.99, 0.999], [0.9, 0.99, 0.999, 0.9999], [0.9, 0.99, 0.999, 0.9999, 0.99999], [0.9, 0.99, 0.999, 0.9999, 0.99999, 0.999999], [0.9, 0.99, 0.999, 0.9999, 0.99999, 0.999999, 0.9999999]

**Table 3:** Default and tuned performance of Transformer-based baselines (10-step prediction).

Models	Setting	RMSE	MAE	SMAPE	$R^{2}$
Transformer	Default	59.80	50.20	7.62	0.99919
	Tuned	55.58	43.66	7.15	0.99937
Informer	Default	70.90	55.40	10.75	0.99895
	Tuned	66.34	51.86	9.48	0.99910
Autoformer	Default	49.20	38.80	6.70	0.99949
	Tuned	47.01	36.60	6.26	0.99955

**Table 4:** Assessment of model accuracy for 10-step prediction (mean ± std over 5 runs).

Group	Models	Model Parameters	RMSE	MAE	SMAPE	$R^{2}$	Improvement (%)
Case 1	CA + Kalman filter [11]	T = 0.5, R = 400, Q = 100	606.70	493.29	11.14	0.36610	98.61%
		T = 1, R = 1600, Q = 200	1434.13	1163.88	27.67	−2.54202	99.41%
	CV + Kalman filter [10]	T = 0.5, R = 400, Q = 100	270.49	225.97	7.40	0.74385	96.88%
		T = 1, R = 1600, Q = 200	339.42	277.60	9.05	0.59667	97.51%
	Singer + Kalman filter [12]	T = 0.5, R = 400, Q = 100, A = 2	301.63	248.38	8.11	0.68148	97.20%
		T = 1, R = 1600, Q = 200, A = 1	401.33	328.43	10.71	0.43612	97.89%
	Current Statistical + Kalman filter [13]	T = 0.1, a = 0.2, R = 25, xamax = 30	242.54	210.14	6.93	0.79405	96.52%
		T = 0.2, a = 0.5, R = 50, xamax = 50	398.16	324.25	10.56	0.44499	97.88%
Case 2	GRU [20]		20.46 ± 3.1	15.95 ± 2.7	0.46 ± 0.12	0.99963 ± 0.0001	58.70%
	LSTM [21]		17.80 ± 2.8	14.72 ± 2.4	0.42 ± 0.11	0.99974 ± 0.0001	52.53%
	CNN-LSTM [23]		24.49 ± 4.4	21.78 ± 3.0	0.64 ± 0.20	0.99973 ± 0.0001	65.50%
	Transformer [24]		55.58 ± 14.2	43.66 ± 7.8	7.15 ± 2.3	0.99937 ± 0.0001	84.80%
	Informer [26]		66.34 ± 17.5	51.85 ± 12.3	9.48 ± 3.1	0.99910 ± 0.0001	87.26%
	Autoformer [27]		47.01 ± 11.5	36.60 ± 7.8	6.26 ± 2.4	0.99955 ± 0.0001	82.03%
	PFVAE [28]		13.02 ± 3.4	10.36 ± 3.1	0.35 ± 0.11	0.99975 ± 0.0001	35.10%
Case 3	SCNs [8]		13.31 ± 4.7	7.49 ± 1.8	0.26 ± 0.08	0.99937 ± 0.0001	36.51%
Proposed	AttSCNs		8.45 ± 1.5	4.64 ± 2.3	0.15 ± 0.05	0.99981 ± 0.0001	

**Table 5:** Assessment of model accuracy for 24-step prediction (mean ± std over 5 runs).

Group	Models	Model Parameters	RMSE	MAE	SMAPE	$R^{2}$	Improvement (%)
Case 1	CA + Kalman filter [11]	T = 0.5, R = 400, Q = 100	2587.78	2091.32	52.36	−10.53103	99.47%
		T = 1, R = 1600, Q = 200	7092.84	5740.58	105.12	−85.62688	99.81%
	CV + Kalman filter [10]	T = 0.5, R = 400, Q = 100	437.96	355.47	8.02	0.66340	96.88%
		T = 1, R = 1600, Q = 200	654.05	534.58	12.09	0.24931	97.91%
	Singer + Kalman filter [12]	T = 0.5, R = 400, Q = 100, A = 2	542.11	442.34	14.48	-0.03427	97.48%
		T = 1, R = 1600, Q = 200, A = 1	831.17	686.48	22.98	−1.43129	98.35%
	Current Statistical + Kalman filter [13]	T = 0.1, a = 0.2, R = 25, xamax = 30	405.71	326.79	10.59	0.42071	96.63%
		T = 0.2, a = 0.5, R = 50, xamax = 50	1025.58	833.57	28.56	−2.70162	98.67%
Case 2	GRU [20]		29.66 ± 5.2	20.59 ± 3.7	0.52 ± 0.15	0.99988 ± 0.0001	53.88%
	LSTM [21]		22.05 ± 3.8	18.49 ± 3.1	0.51 ± 0.13	0.99978 ± 0.0001	37.96%
	CNN-LSTM [23]		25.98 ± 4.1	21.08 ± 3.6	0.55 ± 0.16	0.99990 ± 0.0001	47.34%
	Transformer [24]		65.45 ± 15.7	51.04 ± 9.2	7.65 ± 2.5	0.99912 ± 0.0001	79.10%
	Informer [26]		78.69 ± 18.3	60.11 ± 11.4	12.75 ± 3.2	0.99874 ± 0.0001	82.62%
	Autoformer [27]		88.42 ± 20.4	63.57 ± 12.7	8.04 ± 2.6	0.99841 ± 0.0001	84.53%
	PFVAE [28]		21.65 ± 4.2	18.65 ± 3.3	0.62 ± 0.18	0.99956 ± 0.0001	36.81%
Case 3	SCNs [8]		17.69 ± 3.9	13.08 ± 2.7	0.42 ± 0.13	0.99889 ± 0.0001	22.67%
Proposed	AttSCNs		13.68 ± 2.6	7.63 ± 1.9	0.16 ± 0.06	0.99963 ± 0.0001	

**Table 6:** Ablation of AttSCNs components.

Models	RMSE	MAE	SMAPE	$R^{2}$
SCNs	13.31	7.49	0.26	0.99937
Attention	22.24	22.38	0.37	0.99932
AttSCNs (Default parameters)	9.12	4.86	0.19	0.99965
AttSCNs (After Bayesian optimization)	8.45	4.64	0.15	0.99981

**Table 7:** Comparison of computational efficiency and estimated latency across models.

Model	Parameters	MACs (per Step)	Estimated Latency
GRU	121,400	∼$7.2\times 10^{5}$	1.0–1.5 ms (CPU), <0.2 ms (GPU)
LSTM	161,600	∼$9.5\times 10^{5}$	1.2–1.8 ms (CPU), <0.2 ms (GPU)
CNN-LSTM	168,000	∼$1.1\times 10^{6}$	1.5–2.0 ms (CPU), <0.3 ms (GPU)
Transformer	8,652,800	∼$5.4\times 10^{7}$	15–20 ms (CPU), 1.5–2.0 ms (GPU)
Informer	8,652,800	∼$5.5\times 10^{7}$	16–22 ms (CPU), 1.6–2.2 ms (GPU)
Autoformer	3,293,696	∼$2.1\times 10^{7}$	6.0–8.0 ms (CPU), 0.8–1.2 ms (GPU)
PFVAE	245,000	∼$1.5\times 10^{6}$	2.0–3.0 ms (CPU), <0.4 ms (GPU)
SCNs	132,000	∼$6.8\times 10^{5}$	0.9–1.3 ms (CPU), <0.2 ms (GPU)
AttSCNs	126,252	∼$6.1\times 10^{5}$	0.8–1.2 ms (CPU), <0.1 ms (GPU)

## Data Availability

Data extraction tables can be provided by the authors upon reasonable request.
